# Changes in activity impairment and work productivity after treatment for vitreous hemorrhage due to proliferative diabetic retinopathy: Secondary outcomes from a randomized controlled trial (DRCR Retina Network Protocol AB)

**DOI:** 10.1371/journal.pone.0293543

**Published:** 2023-11-16

**Authors:** Wesley T. Beaulieu, Maureen G. Maguire, Andrew N. Antoszyk, on behalf of the DRCR Retina Network

**Affiliations:** 1 Jaeb Center for Health Research, Tampa, Florida; 2 Charlotte Eye, Ear, Nose, and Throat Associates, Charlotte, North Carolina; Universita degli Studi di Firenze, ITALY

## Abstract

**Background:**

Vitreous hemorrhage from proliferative diabetic retinopathy can cause severe vision loss. DRCR Retina Network Protocol AB was a randomized clinical trial comparing intravitreal aflibercept versus vitrectomy with panretinal photocoagulation and found no difference in the average rate of visual recovery over 104 weeks. Herein, we describe patient-reported outcome measures from Protocol AB.

**Methods:**

Secondary analysis of a multicenter (39 sites) randomized clinical trial. The Work Productivity and Activity Impairment Questionnaire was administered at 4, 12, 24, 36, 52, 68, 84, and 104 weeks. Main outcomes were mean change in activity impairment and work productivity loss over 24 and 104 weeks (area under the curve).

**Results:**

Mean (SD) activity impairment at baseline was 58% (27%) in the aflibercept group (N = 99) and 56% (30%) in the vitrectomy group (N = 105). The mean reduction in activity impairment from baseline over 24 weeks was 21% (25%) in the aflibercept group and 27% (31%) in the vitrectomy group (adjusted difference = -6.8% [95% CI, -12.7% to -0.9%], *P* = .02); over 104 weeks, the adjusted mean difference was -3.1% (95% CI, -9.2% to 3.0%, *P* = .31). Mean work productivity loss at baseline was 51% (28%) in the aflibercept group (N = 44) and 58% (30%) in the vitrectomy group (N = 43). The mean reduction in work productivity loss from baseline over 24 weeks (area under the curve) was 19% (23%) in the aflibercept group and 31% (24%) in the vitrectomy group (adjusted difference = -8.3% [95% CI, -16.8% to 0.2%], *P* = .06); over 104 weeks, the adjusted mean difference was -9.1% (95% CI, -18.4% to 0.2%, *P* = .05).

**Conclusions:**

Participants with vitreous hemorrhage from proliferative diabetic retinopathy had less activity impairment over 24 weeks when treated initially with vitrectomy and panretinal photocoagulation versus intravitreal aflibercept. The trend was similar for work productivity but not statistically significant. By 104 weeks, the improvements were similar in the two treatment groups.

**Trial registration:**

ClinicalTrials.gov NCT02858076.

## Introduction

Patient-reported outcome measures are often collected in clinical trials to incorporate patient perspective into the study results alongside clinical measures. They can be particularly important in a study showing no difference in important clinical outcomes. In ophthalmic studies, patient-reported outcomes are typically more sensitive to vision changes in the better-seeing eye [1].

DRCR Retina Network Protocol AB was a randomized clinical trial of initial treatment with aflibercept versus vitrectomy with panretinal photocoagulation (PRP) for eyes with vitreous hemorrhage (VH) from proliferative diabetic retinopathy (PDR). Enrolled eyes had substantial visual impairment with a median baseline visual acuity (VA) of 20/200 at baseline. Eyes assigned to aflibercept received 4 mandatory, monthly intravitreal injections through week 16 with additional injections through 2 years if retinal neovascularization was present or VH persisted and with vitrectomy if VH causing vision impairment persisted after 1 month post-surgery. Eyes assigned to vitrectomy with PRP had surgery within 2 weeks, performed in a hospital or outpatient surgical center, typically with use of local anesthesia. Eyes assigned to vitrectomy with VH at 4 weeks or later were treated with 2 mandatory injections of aflibercept with additional injections through 2 years if VH failed to resolve.

Mean VA improved substantially in both treatment groups, but the difference in the primary outcome of mean VA over 24 weeks (area under the curve) was not statistically significant (the vitrectomy group had a greater mean improvement by 5.0 letters [95% CI, -0.3 to 10.2]; *P* = .06) [2]. Mean VA was 20/100 in the aflibercept group versus 20/63 in the vitrectomy group at 4 weeks (*P* = .003) and 20/40 in both groups at 104 weeks (*P* = .36), suggesting an early benefit of vitrectomy that waned over follow-up time. One-third of eyes in each group received the alternative treatment per protocol (vitrectomy and PRP in the aflibercept group or aflibercept in the vitrectomy group) when their eye condition worsened or failed to improve during follow-up. Post hoc analyses suggested that eyes with greater baseline vision impairment (worse than 20/800) had faster VA improvement when treated with vitrectomy and PRP compared with aflibercept and that vitrectomy with PRP also resulted in faster clearance of VH than intravitreal aflibercept [3].

Given that there was no significant difference in the primary visual acuity outcome between the aflibercept and vitrectomy groups in Protocol AB, patient-reported outcome measures may be useful in assessing other differences in the effects of these treatments. The Work Productivity and Activity Impairment Questionnaire [4] measures the degree to which patients are impaired by the specific health problem under study in performing everyday activities and in their ability to be productive at work. Herein we present patient-reported outcomes measures from Protocol AB from the Work Productivity and Activity Impairment Questionnaire [4].

## Methods

Methods for Protocol AB (ClinicalTrials.Gov identifier: NCT02858076) have been reported elsewhere [2]. This study adhered to the tenets of the declaration of Helsinki. The ethics board associated with each site provided approval. Study participants provided written informed consent. An independent data and safety monitoring committee provided oversight. Adults with VH from PDR were randomly assigned to initial treatment with intravitreal aflibercept (Eylea, Regeneron Pharmaceuticals, Tarrytown, NY) or vitrectomy with PRP. One eye per participant was enrolled. Eyes in each group could receive the alternative treatment if prespecified criteria were met. The primary outcome was mean VA over 24 weeks (area under the curve calculated using the trapezoidal rule) [2]. Follow-up concluded at 104 weeks.

The Work Productivity and Activity Impairment Questionnaire was used to measure the overall percentage of activity impairment due to VA (a scale from 0% to 100% indicating the level of impairment in performing everyday activities with higher values indicating greater impairment) and work productivity loss (a combination of work time missed and impairment while working) during the prior 7 days leading up to the day on which the questionnaire was administered [4]. The questionnaire and scoring algorithm are available in the online supplement. The questionnaire was interviewer-administered, available in English and Spanish, and administered at baseline, 4, 12, 24, 36, 52, 68, 84, and 104 weeks. Mean change over 24 weeks (weighted average of baseline, 4, 12, and 24 weeks) and over 104 weeks (weighted average of all measures through 104 weeks) in activity impairment and work productivity loss were prespecified secondary outcomes.

Linear regression using the robust sandwich estimator for the variance was used to estimate the treatment group difference and corresponding 95% confidence interval (CI) with adjustment for baseline score (activity impairment or work productivity loss) and lens status per the prespecified statistical analysis plan [5]. All other analyses were conducted post hoc. P values ≤ .05 were considered of interest. Multiple outcomes were evaluated without adjustment for multiplicity; therefore, results should be considered hypothesis-generating. Analyses were conducted with SAS version 9.4 (SAS Institute Inc., Cary, NC).

## Results

### Study cohort

Among the 205 participants enrolled in Protocol AB, 204 (>99%) completed the questionnaire at baseline and provided activity impairment data; 94 of 204 (46%) were employed at baseline, and 87 of 94 (93%) employed participants provided data on work productivity loss. Whether the participant was not working due to vision problems was not collected. Considering the cohort of participants who provided activity impairment data (N = 204), the mean age was 57 years, 89 were women (44%), mean study eye visual acuity was 34 letters (approximate Snellen equivalent 20/200), and mean activity impairment was 57%; most participants self-identified as White (83 [41%]), Hispanic or Latino (83 [41%]), or Black/African American (27 [13%]) (Table 1). Compared with the full cohort, the cohort of participants who provided work productivity data (N = 87) was younger (mean age 52 vs 57 years) and had lower activity impairment (mean 45% vs 57%) (S1 Table). The mean work productivity loss at baseline was 55%.

**Table 1:** Baseline characteristics.

	Treatment Group	
	Aflibercept	Vitrectomy
No. of eyes		
N	99	105
**Participant Characteristics**		
Sex		
Female	46 (46%)	43 (41%)
Male	53 (54%)	62 (59%)
Age, y		
Mean (SD)	56 (12)	57 (11)
Race/Ethnicity		
American Indian/Alaskan Native	1 (1%)	0
Asian	2 (2%)	5 (5%)
Black/African American	16 (16%)	11 (10%)
Hispanic or Latino	42 (42%)	41 (39%)
White	36 (36%)	47 (45%)
More than one race	1 (1%)	0
Unknown/not reported	1 (1%)	1 (<1%)
Diabetes Type		
Type 1	17 (17%)	19 (18%)
Type 2	82 (83%)	86 (82%)
Diabetes Duration, y		
Mean (SD)	19 (11)	21 (11)
Insulin Used		
No	22 (22%)	27 (26%)
Yes	77 (78%)	78 (74%)
Hemoglobin A1c, %		
Mean (SD)	9 (2)	8 (2)
N	95	104
Mean Arterial Pressure, mmHg		
Mean (SD)	102 (12)	102 (12)
Body Mass Index, kg/m^2^		
Mean (SD)	31 (7)	32 (7)
N	84	93
Smoking Status		
Never	60 (61%)	72 (69%)
Prior	26 (26%)	25 (24%)
Current	13 (13%)	8 (8%)
**Ocular Characteristics**		
Lens Status		
PC IOL	25 (25%)	24 (23%)
Phakic	74 (75%)	81 (77%)
Study Eye Visual Acuity		
Letters, Mean (SD)	35 (28)	34 (29)
Approximate Snellen equivalent, Mean	20/200	20/250
20/32 to 20/40 (78 to 69 letters)	16 (16%)	15 (14%)
20/50 to 20/80 (68 to 54 letters)	18 (18%)	24 (23%)
20/100 to 20/160 (53 to 39 letters)	13 (13%)	9 (9%)
20/200 to 20/800 (38 to 4 letters)	26 (26%)	24 (23%)
Worse than 20/800 (≤3 letters)	26 (26%)	33 (31%)
Non-Study Eye Visual Acuity		
Letters, Mean (SD)	71 (21)	74 (17)
Approximate Snellen equivalent, Mean	20/40	20/40
20/25 or better (≥79 letters)	46 (46%)	49 (47%)
20/32 to 20/40 (78 to 69 letters)	26 (26%)	29 (28%)
20/50 to 20/80 (68 to 54 letters)	9 (9%)	17 (16%)
20/100 to 20/160 (53 to 39 letters)	9 (9%)	4 (4%)
20/200 to 20/800 (38 to 4 letters)	6 (6%)	4 (4%)
Worse than 20/800 (≤3 letters)	3 (3%)	2 (2%)
Intraocular Pressure, mmHg		
Mean (SD)	16 (4)	15 (3)
**Work Productivity and Activity Impairment Questionnaire**		
Overall Activity Impairment, %		
Mean (SD)	58 (27)	56 (30)
N	99	105
Work Productivity Loss, %		
Mean (SD)	51 (28)	58 (30)
N	44	43

Abbreviations: PC IOL = posterior chamber intraocular lens, SD = standard deviation.

### Activity impairment

**Fig 1:**
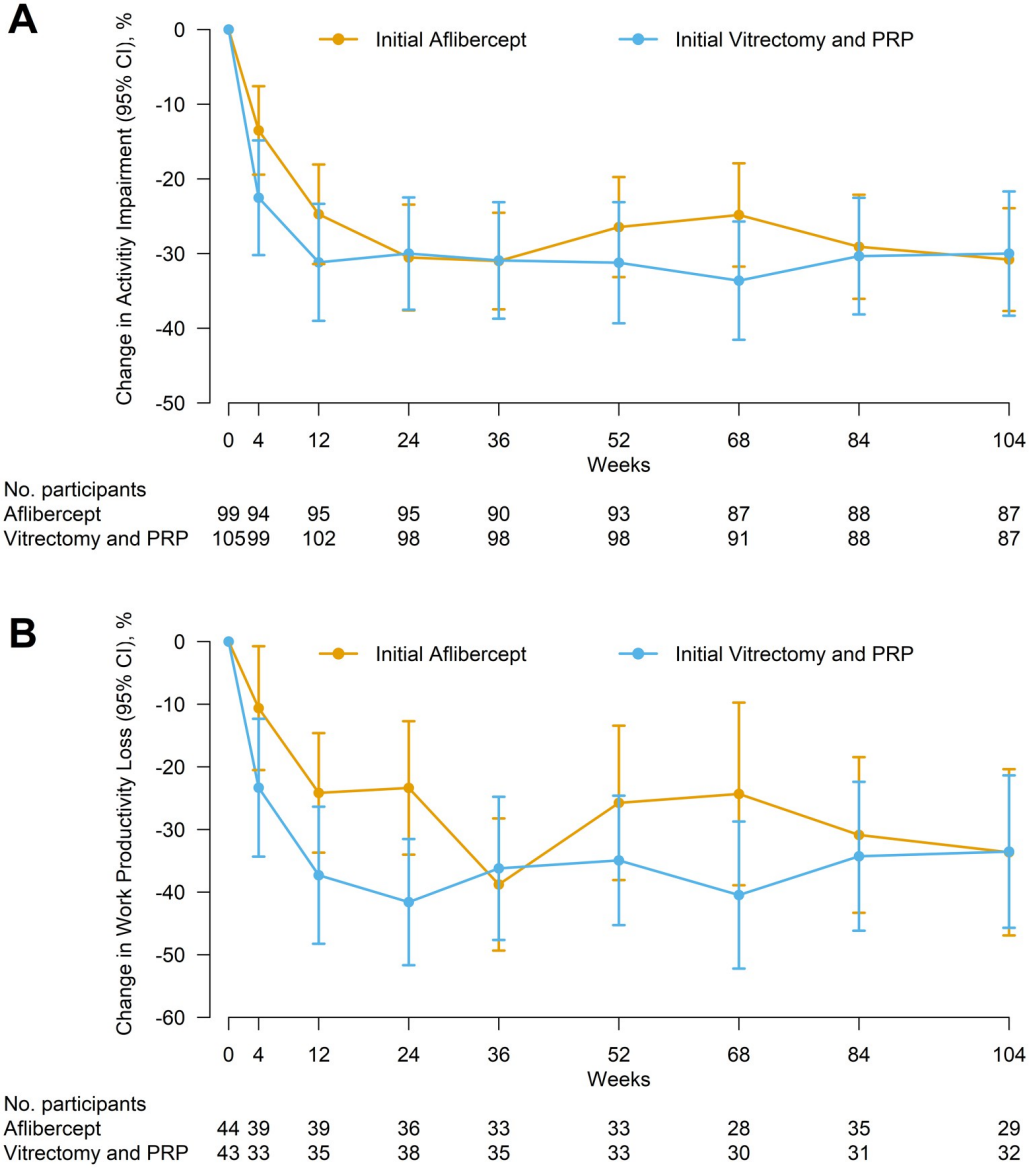
Change in activity impairment and work productivity loss from baseline by treatment group over 104 weeks. Mean change in activity impairment (A) and work productivity loss (B) by treatment group. Error bars represent 95% confidence intervals.

Mean activity impairment at baseline was 58% (SD, 27%) in the aflibercept group (N = 99) and 56% (SD, 30%) in the vitrectomy group (N = 105) (Table 1). The mean change in activity impairment from baseline over 24 weeks (area under the curve) was -21% (SD, 25%) in the aflibercept group (N = 95) and -27% (SD, 31%) in the vitrectomy group (N = 98); the adjusted mean difference was 6.8% (95% CI, 0.9% to 12.7%, *P* = .02; a positive difference indicates greater reduction in activity impairment with vitrectomy compared with aflibercept) (Fig 1A, Table 2). This difference was driven by between-group differences of 9.7% (95% CI, 1.8% to 17.6%, *P* = .02) at 4 weeks and 8.0% (95% CI, -0.1% to 16.1%, *P* = .05) at 12 weeks that waned to 0.5% (95% CI, -7.6% to 8.7%, *P* = .89) at 24 weeks (S2 Table). At 104 weeks, the mean change from baseline in activity impairment was 31% (SD, 32%) in the aflibercept group (N = 87) and 30% (SD, 39%) in the vitrectomy group (N = 87) with an adjusted difference of 1.1% (95% CI, -6.9% to 9.2%, *P* = 78). The adjusted treatment-group mean difference over 104 weeks was 3.1% (95% CI, -3.0% to 9.2%, *P* = .31).

**Table 2:** Activity impairment and work productivity loss at baseline and over 24 and 104 weeks by treatment group.

	Aflibercept	Vitrectomy	Adjusted Mean Difference (95% CI) ^a^	Adjusted Mean Difference (95% CI) ^a^^b^	Adjusted Mean Difference (95% CI) ^a^^b^^c^
Activity impairment at baseline, %					
N	99	105			
Mean (SD)	58 (27)	56 (30)			
Activity impairment area under the curve change from baseline at 24 weeks, %					
N	95	98			
Mean (SD)	-21 (25)	-27 (31)	6.8 (0.9, 12.7)	6.9 (1.1, 12.8)	6.2 (0.4, 12.1)
			p = 0.02	p = 0.02	p = 0.04
Activity impairment area under the curve change from baseline at 104 weeks, %					
N	87	87			
Mean (SD)	-28 (25)	-29 (33)	3.1 (-3.0, 9.2)	3.1 (-2.8, 9.0)	5.0 (-0.8, 10.8)
			p = 0.31	p = 0.30	p = 0.09
Work productivity loss at baseline, %					
N	44	43			
Mean (SD)	51 (28)	58 (30)			
Work productivity loss area under the curve change from baseline at 24 weeks, %					
N	36	38			
Mean (SD)	-19 (23)	-31 (24)	8.3 (-0.2, 16.8)	9.0 (0.8, 17.2)	9.4 (1.6, 17.3)
			p = 0.06	p = 0.03	p = 0.02
Work productivity loss area under the curve change from baseline at 104 weeks, %					
N	29	32			
Mean (SD)	-25 (27)	-35 (27)	9.1 (-0.2, 18.4)	8.8 (-0.1, 17.7)	5.8 (-2.0, 13.6)
			p = 0.05	p = 0.05	p = 0.14

Abbreviations: SD = standard deviation.

^a^ Adjusted for baseline score and lens status.

^b^ Adjusted for baseline visual acuity of the better-seeing eye (post hoc).

^c^ Longitudinal linear mixed model to handle missing data via maximum likelihood (post hoc); repeated measurements on participants modeled using an unstructured covariance matrix and clustering by clinical site modeled with random intercepts.

**Fig 2:**
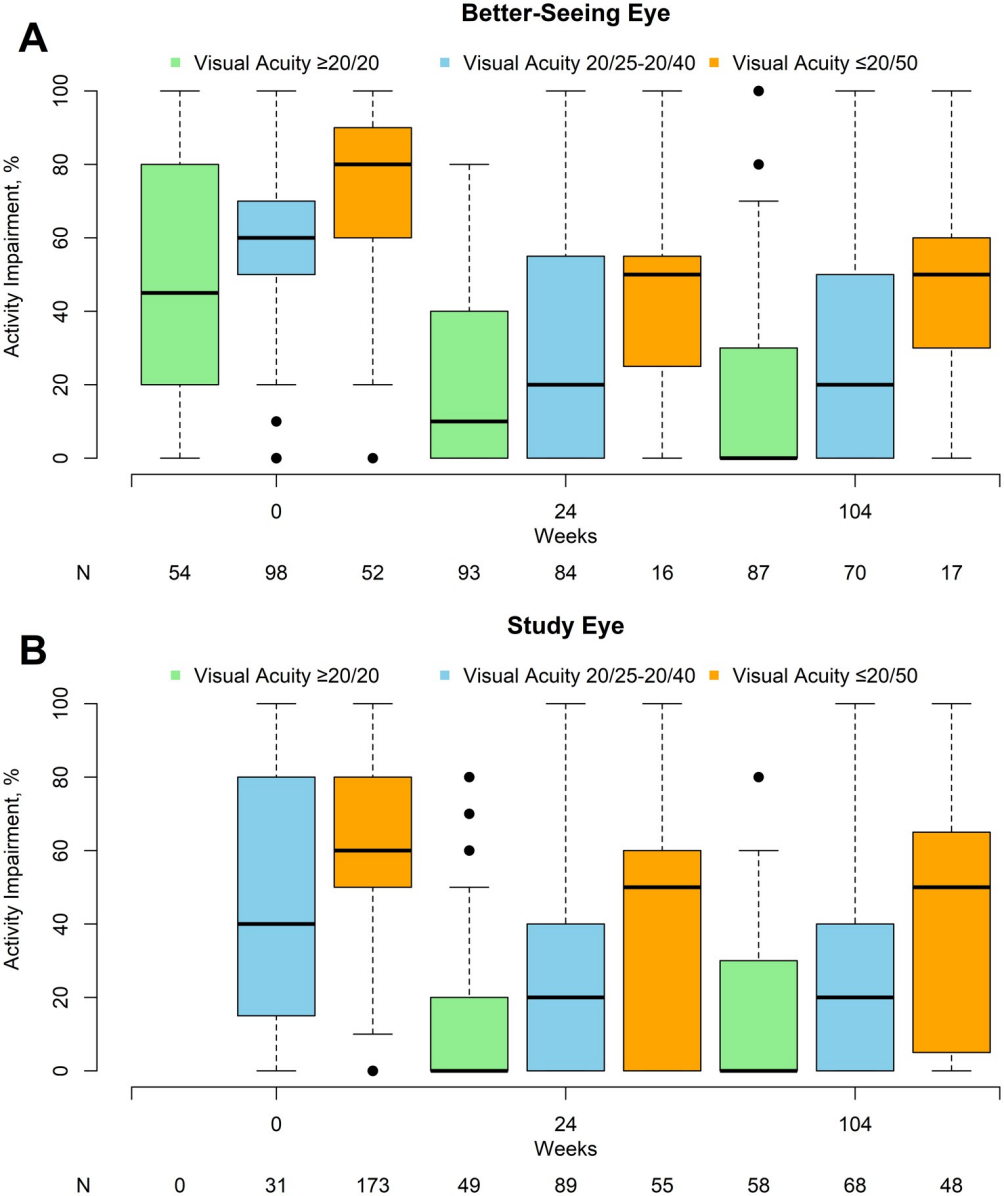
Activity impairment by visual acuity in the better-seeing and study eye at baseline, 24, and 104 weeks. Boxplot of activity impairment at baseline, 24, and 104 weeks by visual acuity in the (A) better-seeing eye and (B) study eye.

**Fig 3:**
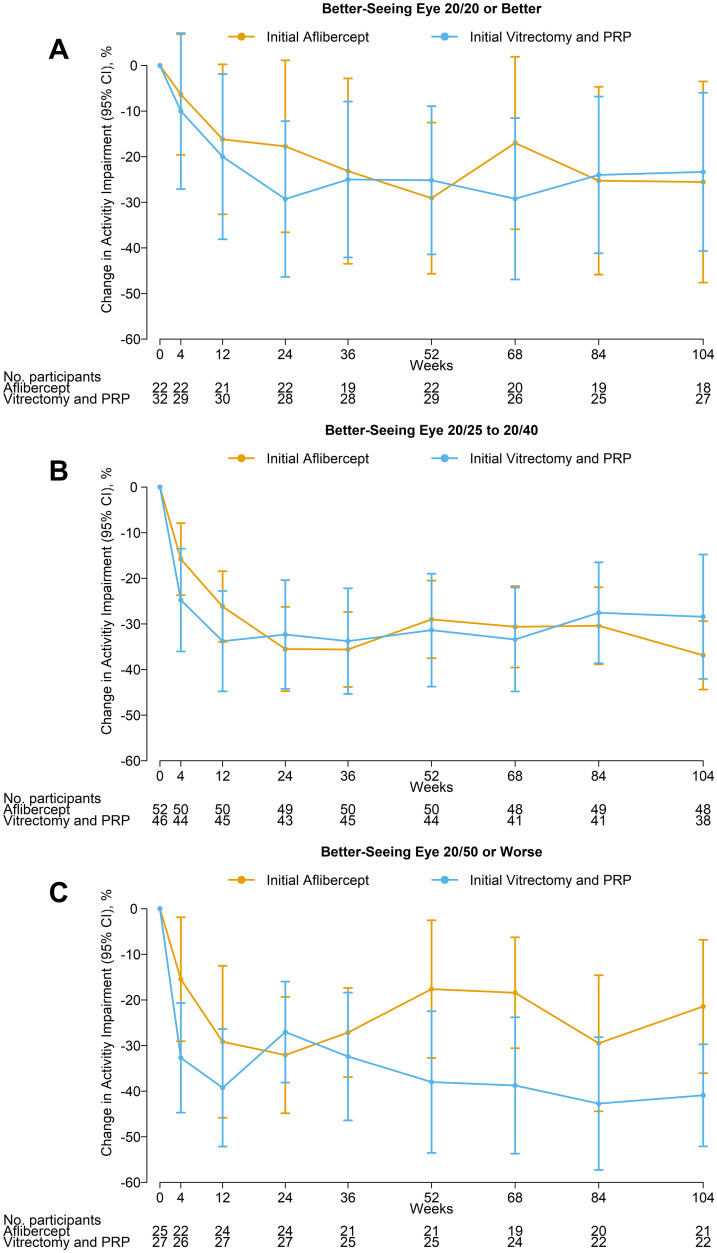
Activity impairment by visual acuity in the better-seeing eye at baseline. Mean change in activity impairment from baseline over 104 weeks by baseline visual acuity in the better-seeing eye. Error bars represent 95% confidence intervals.

The mean level of baseline activity impairment was related to the visual acuity in the better-seeing eye: mean activity impairment was 46% when VA in the better-seeing eye was 20/20 or better (N = 54), 56% when 20/25 to 20/40 (N = 98), and 72% when 20/50 or worse (N = 52; Fig 2A). While the level of impairment decreased over time, the relative degree of impairment was still sensitive to the visual acuity in the better seeing eye: mean activity impairment at 104 weeks was 18%, 29%, and 49% when VA in the better-seeing eye was 20/20 or better (N = 87), 20/25 to 20/40 (N = 70), and 20/50 or worse (N = 17), respectively. The study eye was the better-seeing eye in only 13 of 204 eyes (6%) at baseline, which increased to 66 of 193 (34%) at 24 weeks and 74 of 174 (43%) at 104 weeks. Change in activity impairment through 104 weeks by treatment group and baseline VA in the better-seeing eye at baseline is shown in Fig 3. There was no significant interaction between baseline VA in the better-seeing eye and treatment group for change in activity impairment at 104 weeks (*P* = .49) or over 104 weeks (area under the curve, *P* = .69). Adjusting for baseline VA of the better-seeing eye resulted in similar point estimates but slightly narrower confidence intervals (Table 2). A longitudinal model that accounts for missing follow-up data produced similar results (Table 2).

There was a similar relationship between study eye VA and activity impairment. At baseline, the mean activity impairment was 45% when study-eye VA was 20/25 to 20/40 (N = 31) and 59% when 20/50 or worse (N = 173); at 104 weeks, the mean activity impairment was 14%, 25%, and 41% when study eye VA was 20/20 or better (N = 58), 20/25 to 20/40 (N = 68), or 20/50 or worse (N = 48), respectively (Fig 2B). There was no significant interaction between baseline VA in the study eye and treatment group for change in activity impairment at 104 weeks (*P* = .93) or over 104 weeks (area under the curve, *P* = .62).

### Work productivity loss

Among the 87 participants providing work productivity data at baseline, the mean activity impairment was 45% in both groups, which was lower than the full cohort (S1 Table). Mean work productivity loss at baseline was 51% (SD, 28%) in the aflibercept group (N = 44) and 58% (SD, 30%) in the vitrectomy group (N = 43) (Table 1). The mean change in work productivity loss from baseline over 24 weeks (area under the curve) was -19% (SD, 23%) in the aflibercept group (N = 36) and -31% (SD, 24%) in the vitrectomy group (N = 38); the adjusted mean difference was 8.3% (95% CI, -0.2% to 16.8%, *P* = .06; a positive difference indicates greater reduction in work productivity loss with vitrectomy than aflibercept) (Fig 1B, Table 2). The adjusted treatment group mean difference in work productivity loss was 9.5% (95% CI, -3.3% to 22.3%, *P* = .14) at 4 weeks, 9.2% (95% CI, -2.3% to 20.7%, *P* = .11) at 12 weeks, and 13.9% (95% CI, 2.4% to 25.5%, *P* = .02) at 24 weeks (S2 Table). At 104 weeks, the mean change from baseline in work productivity loss was -34% in both the aflibercept (N = 29) and vitrectomy (N = 32) groups with an adjusted difference of -1.3%% (95% CI, -15.6% to 13.0%, *P* = .86). The adjusted treatment-group difference over 104 weeks (area under the curve) was 9.1% (95% CI, -0.2% to 18.4%, *P* = .05) favoring vitrectomy. After adjusting for baseline VA of the better-seeing eye, the difference of mean change in work productivity loss over 24 weeks was 9.0% (95% CI, 0.8% to 17.2%, *P* = .03) in favor of vitrectomy (Table 2).

At 24 weeks and among the 87 participants contributing work productivity loss data at baseline, 74 (85%) provided data at the 24-week visit, 3 (4%) missed the visit, and 10 (11%) completed the visit and provided activity impairment data but not work productivity loss data. Similarly, at 104 weeks and among the 87 participants contributing work productivity loss data at baseline, 61 (70%) provided work productivity loss data, 8 (9%) missed the visit, and 18 (21%) completed the visit and provided activity impairment data but not work productivity loss data. It is unknown whether the participants who completed the visits but did not provide work productivity loss data were still working and declined to answer the questionnaire or if they had stopped working and, if so, whether their vision was the primary reason for stopping working. A longitudinal model that accounts for missing follow-up data under the missing at random assumption produced similar results to the complete case analyses (Table 2, S2 Table).

## Discussion

In DRCR Retina Network Protocol AB, which compared initial treatment for VH due to PDR with intravitreal aflibercept versus vitrectomy with PRP, there was greater reduction in activity impairment over 24 weeks in the vitrectomy with PRP group compared with the aflibercept group. The mean change in work productivity loss from baseline over 24 weeks suggested a benefit favoring the vitrectomy group, but the difference was not statistically significant until after adjustment for baseline VA in the better-seeing eye. Vision-related quality of life is often sensitive to the VA of the better-seeing eye, which was also the case in Protocol AB.

The mean baseline levels of activity impairment and work productivity loss among Protocol AB patients were similar to the levels seen in patients with rheumatoid or psoriatic arthritis and nearly twice as great as for patients that have PDR without visually-significant VH at baseline [6–8]. In psoriatic arthritis, meaningful long-term reductions in activity impairment and work productivity for an individual have been determined to be 20% and 15%, respectively; [8] in Protocol AB, the long-term reductions in mean activity impairment and work productivity were substantial (approximately 25–35%) and exceeded these thresholds. These reductions in activity impairment and work productivity loss coincided with substantial VA gains in both groups. At 4 weeks, VA was better in the vitrectomy group compared with the aflibercept group, which may explain the significant improvement in activity impairment in the vitrectomy group over the first 24 weeks [2]. Despite these improvements, most participants (108 of 175 [62%]) still reported some degree of activity impairment at 104 weeks and the mean level of activity impairment was 26% overall.

In a post hoc analysis of Protocol AB, eyes with a baseline VA of 20/800 had greater VA improvement over 24 weeks when treated with vitrectomy compared with aflibercept, while there was no difference between groups among eyes with baseline VA of 20/32 to 20/160 [3]. We did not observe a similar interaction between baseline visual acuity or better-seeing eye and treatment group in the analysis of activity impairment or work productivity loss.

As discussed in prior publications, additional factors will affect the choice of treatment for VH from PDR. These include the cost of treatment, access to a surgical center, patient comorbidities, presence of concomitant diabetic macular edema, and the patient’s desire for rapid vision restoration, particularly for those whose affected eye is (or was) their better-seeing eye. Of note, these was no difference in the rate of cataract extraction between groups through 104 weeks [3], and retinal detachments were less frequent in the vitrectomy group [2].

This study has limitations. First, the number of participants measured for work productivity loss was relatively small (87 vs 204 for activity impairment), which limits the power for detecting differences in these outcomes. Second, the Work Productivity and Activity Impairment Questionnaire does not measure whether the reason for not working (or no longer working if the participants was working at baseline) is vision related. Third, approximately 1 in 3 eyes from each group received the alternative treatment (aflibercept or vitrectomy with PRP) per protocol, which likely contributed to the similar outcomes at 104 weeks; however, this was per protocol, and the study was designed to mimic clinical care patterns to enhance applicability in practice. Fourth, because questionnaires were not administered between baseline and 4 weeks, the perioperative impacts of vitrectomy are not reflected in these data. Fifth, the clinical staff administering the questionnaire were not masked to the participant’s treatment assignment; however, staff were instructed to read the questions verbatim. Due to the randomized nature of the trial and high retention (95% through 24 weeks and 90% through 104 weeks) [2], allocation bias and attrition bias should be minimal.

In conclusion, participants with VH from PDR had less impairment when performing everyday activities over 24 weeks when treated initially with vitrectomy and PRP compared with intravitreal aflibercept. The trend was similar for work productivity loss but did not reach significance without further adjustment for VA in the better-seeing eye. By 104 weeks, the improvements were similar.

## Supporting information

S1 Table Baseline characteristics among participants contributing work productivity loss data. (DOCX)

S2 Table Activity impairment and work productivity loss by treatment group. (DOCX)
