# Supplementary material for: Changes in activity impairment and work productivity after treatment for vitreous hemorrhage due to proliferative diabetic retinopathy: Secondary outcomes from a randomized controlled trial (DRCR Retina Network Protocol AB)
Source: PLoS One. 2023 Nov 16;18(11):e0293543. doi: 10.1371/journal.pone.0293543 (PMC10653538; doi:10.1371/journal.pone.0293543)
Supplement: S1 Table — (DOCX) [file pone.0293543.s001.docx]

# Supporting Information Table A. Baseline Characteristics Among Participants Contributing Work Productivity Loss Data

|  | **Treatment Group** | |
| --- | --- | --- |
|  | **Aflibercept** | **Vitrectomy** |
|  | | |
| No. of eyes | | |
| N | 44 | 43 |
|  | | |
| **Participant Characteristics** | | |
| Sex | | |
| Female | 18 (41%) | 15 (35%) |
| Male | 26 (59%) | 28 (65%) |
| Age, y | | |
| Mean (SD) | 52 (10) | 53 (9) |
| Race/Ethnicity | | |
| Asian | 1 (2%) | 3 (7%) |
| Black/African American | 9 (20%) | 4 (9%) |
| Hispanic or Latino | 16 (36%) | 20 (47%) |
| White | 17 (39%) | 15 (35%) |
| More than one race | 1 (2%) | 0 |
| Unknown/not reported | 0 | 1 (2%) |
| Diabetes Type | | |
| Type 1 | 12 (27%) | 6 (14%) |
| Type 2 | 32 (73%) | 37 (86%) |
| Diabetes Duration, y | | |
| Mean (SD) | 20 (10) | 19 (9) |
| Insulin Used | | |
| No | 8 (18%) | 16 (37%) |
| Yes | 36 (82%) | 27 (63%) |
| Hemoglobin A1c, % | | |
| Mean (SD) | 9 (2) | 8 (2) |
| N | 42 | 42 |
| Mean Arterial Pressure, mmHg | | |
| Mean (SD) | 104 (12) | 102 (13) |
| N | 44 | 43 |
| Body Mass Index, kg/m^2^ | | |
| Mean (SD) | 31 (6) | 32 (7) |
| Smoking Status | | |
| Never | 27 (61%) | 31 (72%) |
| Prior | 12 (27%) | 9 (21%) |
| Current | 5 (11%) | 3 (7%) |
|  | | |
| **Ocular Characteristics** | | |
| Lens Status | | |
| PC IOL | 4 (9%) | 4 (9%) |
| Phakic | 40 (91%) | 39 (91%) |
| Study Eye Visual Acuity | | |
| Letters, Mean (SD) | 39 (28) | 36 (29) |
| Approximate Snellen equivalent, Mean | 20/160 | 20/200 |
| 20/32 to 20/40 (78 to 69 letters) | 9 (20%) | 7 (16%) |
| 20/50 to 20/80 (68 to 54 letters) | 7 (16%) | 12 (28%) |
| 20/100 to 20/160 (53 to 39 letters) | 8 (18%) | 2 (5%) |
| 20/200 to 20/800 (38 to 4 letters) | 9 (20%) | 10 (23%) |
| Worse than 20/800 (≤3 letters) | 11 (25%) | 12 (28%) |
| Non-Study Eye Visual Acuity | | |
| Letters, Mean (SD) | 76 (15) | 75 (18) |
| Approximate Snellen equivalent, Mean | 20/32 | 20/32 |
| 20/25 or better (≥79 letters) | 23 (52%) | 23 (53%) |
| 20/32 to 20/40 (78 to 69 letters) | 14 (32%) | 11 (26%) |
| 20/50 to 20/80 (68 to 54 letters) | 1 (2%) | 5 (12%) |
| 20/100 to 20/160 (53 to 39 letters) | 4 (9%) | 1 (2%) |
| 20/200 to 20/800 (38 to 4 letters) | 2 (5%) | 2 (5%) |
| Worse than 20/800 (≤3 letters) |  | 1 (2%) |
| Intraocular Pressure, mmHg | | |
| Mean (SD) | 16 (4) | 15 (3) |
|  | | |
| **Work Productivity and Activity Impairment Questionnaire** | | |
| Overall Activity Impairment, % | | |
| Mean (SD) | 45 (28) | 45 (29) |
| Work Productivity Loss, % | | |
| Mean (SD) | 51 (28) | 58 (30) |
|  | | |

Abbreviations: PC IOL = posterior chamber intraocular lens, SD = standard deviation.
