# Supplementary material for: Changes in activity impairment and work productivity after treatment for vitreous hemorrhage due to proliferative diabetic retinopathy: Secondary outcomes from a randomized controlled trial (DRCR Retina Network Protocol AB)
Source: PLoS One. 2023 Nov 16;18(11):e0293543. doi: 10.1371/journal.pone.0293543 (PMC10653538; doi:10.1371/journal.pone.0293543)
Supplement: S2 Table — (DOCX) [file pone.0293543.s002.docx]

# Supporting Information Table B. Activity Impairment and Work Productivity Loss by Treatment Group

|  | **Aflibercept** | **Vitrectomy** | **Adjusted Mean Difference (95% CI) ^a^** | **Adjusted Mean Difference (95% CI) ^ab^** | **Adjusted Mean Difference (95% CI) ^abc^** |
| --- | --- | --- | --- | --- | --- |
| Change in activity impairment from baseline at 4 weeks, % | | | | |  |
| N | 94 | 99 |  |  |  |
| Mean (SD) | -14 (29) | -23 (39) | 9.7 (1.8, 17.6) | 9.9 (1.9, 17.8) | 9.6 (1.6, 17.7) |
|  |  |  | p = 0.02 | p = 0.01 | p = 0.02 |
|  | | | | |  |
| Change in activity impairment from baseline at 12 weeks, % | | | | |  |
| N | 95 | 102 |  |  |  |
| Mean (SD) | -25 (33) | -31 (40) | 8.0 (-0.1, 16.1) | 8.0 (-0.1, 16.1) | 8.7 (0.4, 17.1) |
|  |  |  | p = 0.05 | p = 0.05 | p = 0.04 |
|  | | | | |  |
| Change in activity impairment from baseline at 24 weeks, % | | | | |  |
| N | 95 | 98 |  |  |  |
| Mean (SD) | -31 (35) | -30 (37) | 0.5 (-7.6, 8.7) | 0.9 (-6.9, 8.7) | 0.7 (-7.1, 8.6) |
|  |  |  | p = 0.89 | p = 0.82 | p = 0.85 |
|  | | | | |  |
| Change in activity impairment from baseline at 52 weeks, % | | | | |  |
| N | 93 | 98 |  |  |  |
| Mean (SD) | -26 (33) | -31 (40) | 5.8 (-2.4, 14.0) | 6.3 (-1.6, 14.1) | 6.4 (-1.6, 14.3) |
|  |  |  | p = 0.16 | p = 0.12 | p = 0.12 |
|  | | | | |  |
| Change in activity impairment from baseline at 104 weeks, % | | | | |  |
| N | 87 | 87 |  |  |  |
| Mean (SD) | -31 (32) | -30 (39) | 1.1 (-6.9, 9.2) | 1.1 (-6.7, 8.9) | 2.8 (-5.1, 10.6) |
|  |  |  | p = 0.78 | p = 0.78 | p = 0.49 |
|  | | | | |  |
| Change in work productivity loss from baseline at 4 weeks, % | | | | |  |
| N | 39 | 33 |  |  |  |
| Mean (SD) | -11 (30) | -23 (31) | 9.5 (-3.3, 22.3) | 11.1 (-1.9, 24.1) | 9.7 (-3.3, 22.6) |
|  |  |  | p = 0.14 | p = 0.09 | p = 0.14 |
|  | | | | |  |
| Change in work productivity loss from baseline at 12 weeks, % | | | | |  |
| N | 39 | 35 |  |  |  |
| Mean (SD) | -24 (29) | -37 (32) | 9.2 (-2.3, 20.7) | 10.2 (-0.6, 21.1) | 8.4 (-2.7, 19.6) |
|  |  |  | p = 0.11 | p = 0.06 | p = 0.14 |
|  | | | | |  |
| Change in work productivity loss from baseline at 24 weeks, % | | | | |  |
| N | 36 | 38 |  |  |  |
| Mean (SD) | -23 (31) | -42 (31) | 13.9 (2.4, 25.5) | 14.7 (3.4, 26.0) | 14.0 (2.5, 25.5) |
|  |  |  | p = 0.02 | p = 0.01 | p = 0.02 |
|  | | | | |  |
| Change in work productivity loss from baseline at 52 weeks, % | | | | |  |
| N | 33 | 33 |  |  |  |
| Mean (SD) | -26 (35) | -35 (29) | 8.5 (-3.0, 20.0) | 9.5 (-1.6, 20.6) | 8.3 (-2.9, 19.5) |
|  |  |  | p = 0.14 | p = 0.09 | p = 0.14 |
|  | | | | |  |
| Change in work productivity loss from baseline at 104 weeks, % | | | | |  |
| N | 29 | 32 |  |  |  |
| Mean (SD) | -34 (35) | -34 (34) | -1.3 (-15.6, 13.0) | -1.8 (-15.4, 11.7) | -4.0 (-17.4, 9.4) |
|  |  |  | p = 0.86 | p = 0.79 | p = 0.56 |
|  | | | | |  |

Abbreviations: CI = confidence interval, SD = standard deviation.

^a^ Adjusted for baseline score and lens status

^b^ Adjusted for baseline visual acuity of the better-seeing eye (post hoc)

^c^ Longitudinal linear mixed model to handle missing data via maximum likelihood (post hoc); repeated measurements on participants modeled using an unstructured covariance matrix and clustering by clinical site modeled with random intercepts.
